# Dietary Inclusion of Dried Chicory Root Affects Cecal Mucosa Proteome of Nursery Pigs

**DOI:** 10.3390/ani12131710

**Published:** 2022-07-01

**Authors:** Agnieszka Herosimczyk, Adam Lepczyński, Martyna Werkowska, Marcin Barszcz, Marcin Taciak, Anna Tuśnio, Andrzej Krzysztof Ciechanowicz, Magdalena Kucia, Karolina Susfał, Sandra Cabała, Małgorzata Ożgo

**Affiliations:** 1 Department of Physiology, Cytobiology and Proteomics, Faculty of Biotechnology and Animal Husbandry, West Pomeranian University of Technology Szczecin, Klemensa Janickiego 29, 71-270 Szczecin, Poland; adam.lepczynski@zut.edu.pl (A.L.); martyna.werkowska@gmail.com (M.W.); susfal_karolina@wp.pl (K.S.); cabala.sandra@op.pl (S.C.); malgorzata.ozgo@zut.edu.pl (M.O.); 2 Department of Animal Nutrition, The Kielanowski Institute of Animal Physiology and Nutrition, Polish Academy of Sciences, Instytucka 3, 05-110 Jabłonna, Poland; m.barszcz@ifzz.pl (M.B.); m.taciak@ifzz.pl (M.T.); a.tusnio@ifzz.pl (A.T.); 3 Laboratory of Regenerative Medicine, Centre for Preclinical Research and Technology, Medical University of Warsaw, Zwirki and Wigury 61, 02-091 Warsaw, Poland; andrzej.ciechanowicz@wum.edu.pl (A.K.C.); magdalena.kucia@wum.edu.pl (M.K.)

**Keywords:** piglet, cecum, mucosa, proteins, prebiotics, chicory inulin, 2-DE map

## Abstract

**Simple Summary:**

A well-balanced diet seems to play a key role in disease prevention and health promotion in young animals. Therefore, many attempts have been made to supplement feeds with novel nutritional components, with potential prebiotic capacity. It seems that chicory root fulfils those criteria as it contains high amounts of inulin-type fructans. Hence, the aim of the study was to determine the effect of dietary supplementation with 4% dried chicory root on the cecal mucosa proteome of piglets. It is shown that this feed additive may affect cellular metabolism in the cecal epithelium and may be beneficial for gut health.

**Abstract:**

Prebiotics are known to have many beneficial effects on intestinal health by modulating the gut microbiota composition, thereby affecting epithelial cell proliferation and metabolism. This study had two aims: (1) to identify the protein constituents in the cecal mucosa of 50-day-old healthy (PIC × Penarlan P76) barrows, and (2) to assess the effects of 4% inclusion of dried chicory root in a cereal-based diet on the cecal mucosa proteome changes. Pigs (eight per group) were randomly allotted to the groups and were fed a control diet from the tenth day of life (C) or a diet supplemented with 4% of died chicory root (CR), for 40 days. At the age of 50 days, animals were sacrificed and cecal tissue samples were collected. It was found that feeding a CR diet significantly decreased the expression of 16 cecal mucosa proteins. Among them, fifteen proteins were down-regulated, while only one (KRT20) was shown to be up-regulated when compared to the C group. Dietary supplementation with CR caused down-expression of metabolism-associated proteins including enzymes involved in the process of glycolysis (G6PD, TPI1, ALDH9A1, CKMT1 and AKR1A1) as well as those engaged in transcriptional and translational activity (PRPF19, EEF1G) and several structural proteins (ACTR3, KRT77, CAP1 and actin). From our findings, it is possible to conclude that dietary chicory root at 4% had beneficial effects on the gut health of pigs as indicated by a changed abundance of certain cecal proteins such as KRT20, SERPINB1, HSP27, ANAXA2 and ANAXA4.

## 1. Introduction

Considering its remarkably diverse and complex network of cell populations per-forming a wide variety of functions including digestion, absorption, secretion and immunological defense, the gastrointestinal tract (GIT) is believed to be one of the most multifunctional organ systems [1]. In addition, the GIT provides an effective frontline barrier against invading pathogens and other potentially harmful agents, and most importantly, it harbors a complex community of microorganisms, collectively known as the GI microbiota [1]. Owing to suitable environmental conditions, such as slow digesta passage, neutral pH and low redox potential, the large intestine of pigs (*Sus scrofa*) is the predominant colonization site for large and diverse populations of commensal microbiota [2,3]. 

There are several intrinsic and extrinsic determinants of gut health, the most important of which include diet, effective digestion and nutrient absorption, intestinal permeability, gut microbiota, and the proper functioning of the immune system [4]. Among the above-mentioned factors, a well-balanced diet seems to play a key role in disease prevention and health promotion. Therefore, many attempts have been made to supplement feeds with novel nutritional components with potential prebiotic capacity. The root of chicory (*Cichorium intybus* L.)—a plant rich in fiber—seems to meet these criteria, because it contains high contents of inulin and fructo-oligosaccharides (FOS) [5]. As reviewed by Mensink et al. [6], the unique properties of inulin result from its chemical structure, which prevents it from being digested in the upper parts of the GIT, where it remains intact until hydrolyzed by microbial enzymes. Short-chain fatty acids (SCFAs), the primary end products of bacterial fermentation of inulin, exert local effects in the gut (predominantly butyrate) [7]. Nevertheless, a large portion of SCFAs are transported from the intestinal lumen into the bloodstream (mainly acetate and propionate), where they can act as both energetic substrates and signal molecules in many tissues and organs [7]. This is further supported by the results of our previous studies, where dietary chicory root has been shown to exert multiple systemic effects, including expression changes of proteins involved in key metabolic pathways in different porcine tissue/organ systems [8,9] as well as biological fluids [10]. Various studies have clearly demonstrated that the prebiotic effect is highly associated with both inulin chain length (degree of polymerization, DP) and its dietary level. DP refers to the number of individual monosaccharide units in the polysaccharide chain [6]. Such effects were observed in the large intestine, mainly in the cecum of pigs fed diets supplemented with inulin (1–4%), with an average DP of 25 [11,12] or 23 [13]. On the other hand, Paßlack et al. [14] conducted a study on weaned piglets and showed that dietary supplementation with 4% long-chain inulin (DP ≤ 57) led to an increase in *Lactobacillus* abundance only in the proximal part of the colon. Another experiment, carried out on growing piglets, showed that the addition of 3% inulin to the feed with an average DP of 12 caused its significant degradation (20–50%) in the jejunum [15].

In addition, chicory root contains other phytochemicals that are collectively known to have antioxidant, anticancer and anti-inflammatory properties [16]. These bioactive substances include coumarins, flavonoids, tannins, alkaloids, vitamins, minerals and essential oils [16]. One of our recent studies also demonstrated that the inclusion of chicory in a cereal-based diet could significantly influence kidney and liver mineral contents and antioxidant capacity in nursery pigs [17]. It is currently believed that there is a signaling crosstalk between the epithelium, mucosal immune system and gut microbial ecosystem, which is crucial for maintaining intestinal epithelial cell homeostasis, and thus animal health and performance [18]. The gut microbiota can produce a large number of biologically active metabolites that predominantly originate from prebiotic dietary sources, including SCFAs. These metabolic end products have been shown to directly affect epithelial cell metabolism and gut barrier function by up-regulating adhesive junctional proteins [19]. In a previous study, we explored the effect of dietary inclusion of 1 and 3% native inulin on the proteome of large intestinal mucosa (cecum, proximal and distal colon) in nursery pigs [20]. The results of this work clearly show that a diet supplemented with 3% inulin caused a significant up-regulation of vinculin—a protein that forms one of the junctional complexes—in the distal colon [20].

Studies aimed at identifying the proteome composition in the cecal mucosa of healthy nursery pigs are still scarce. Therefore, one of the objectives of the present study was to establish the first 2-DE-based protein map of the porcine cecal mucosa. Although Barmpatsalou et al. [21] have recently presented a comprehensive physiological and structural characterization of mucus in the entire porcine GIT, from the stomach to the colon, this large-scale study was carried out in adult pigs (20–22 weeks of age). Given the age-related changes in the physiology and function of the gut, our results may complement the existing data on the main features of the mucosal protective barrier in the cecum of nursery pigs at the protein level. 

We hypothesized that a 40-day dietary supplementation with 4% dried chicory root, as a rich source of inulin and FOS, could enhance the barrier function of the cecal mucosa by increasing the concentration of cecal tight junction proteins, as well as those displaying cytoprotective, metabolic and antioxidant properties. Considering the above, we employed two-dimensional electrophoresis (2-DE), followed by matrix-assisted laser desorption/ionization (MALDI), time-of-flight (TOF) and MALDI Fourier-transform ion cyclotron resonance (FT-ICR) mass spectrometry to (1) identify the protein constituents in the cecal mucosa of 50-day-old healthy barrows and to (2) gain insight into the effect of dietary supplementation with 4% dried chicory root on the proteome of the cecal mucosa in pigs.

## 2. Materials and Methods

### 2.1. Animals, Study Design and Sample Collection

The experiment was performed on 16 crossbred (PIC × Penarlan P76) barrows divided into two groups (*n* = 8), offered cereal-based pre-starter diets from the 10th day of life: control diet (C) or a diet with 4% addition of dried chicory root (CR) (VITAMIX, Niemcz, Poland). The ingredient composition as well as nutrient and energy content of the diets (Table 1), as well as the chemical composition of chicory root (Table 2) were previously described by Lepczyński et al. [17]. Chemical analysis of feeds (dry matter, crude ash, crude protein, ether extract, crude fibre and fructan content) was determined according to the methods of AOAC [22].

From birth until weaning, the piglets were kept with their sows (of third and fourth parity) in farrowing pens on a commercial farm (4 sows with their litters per group). During the first 9 days of life, piglets received only colostrum and milk from their mothers, but at day 10 of age, they were also offered the experimental diets as a mash placed in a feeder. Feed intake was not measured in this period. 

At day 28 of age, piglets were weaned and weighed. Based on body weight, two barrows from each litter were chosen, ear-tagged, and transported to the experimental facility, where they were divided according to dietary treatment and placed in pens of 4 piglets each (2 pens per group). After weaning, experimental diets were given in the form of pellets of 4 mm diameter. Animals had free access to feed and water throughout the whole experimental period. After 40 days of feeding, at the age of 50 days pigs were euthanized by electric stunning and exsanguinated.

The gastrointestinal tract was removed immediately after animal sacrifice to collect tissue samples from the cecum. Dissected specimens were washed twice with 0.9% NaCl and thereafter twice with 20 mM Krebs-HEPES buffer (99 mM NaCl, 4.69 mM KCl, 2.50 mM CaCl_2_, 1.20 mM MgSO_4_, 1.03 mM K_2_HPO_4_, 25 mM NaHCO_3_, 5.60 mM D-(+)-glucose, pH 7.4). Upon collection, tissue samples were snap-frozen in liquid nitrogen and stored at −80 °C until further analysis. The experimental procedures were approved by the Polish Local Commission of Ethics for the Care and Use of Laboratory Animals (No. 13/2012 of 23 May 2012).

### 2.2. Two-Dimensional Electrophoresis (2-DE)

Cecal mucosal surfaces were gently scrubbed and homogenized in 1000 µL of lysis buffer containing 7 M urea, 2 M thiourea, 4% *w*/*v* CHAPS, 1% *w*/*v* DTT, 2% *v*/*v* Biolyte, 1% *v*/*v* protease inhibitor cocktail and 0.1% *v*/*v* nuclease at a frequency of 20,000 Hz for 60 min using a mechanical homogenizer (Tissue Lyser, QIAGEN, Hilden, Germany). Insoluble tissue debris was removed by centrifugation (4 °C, 15,000× *g*, 15 min) and the supernatant was used for 2-DE.

For isoelectric focusing (IEF), 1000 µg of protein was mixed with a lysis buffer up to 650 µL total volume and applied to 3–10 24 cm ReadyStrip™ IPG Strips (Bio-Rad, Hercules, CA, USA). IEF was performed using Protean^®^ IEF Cell (Bio-Rad, Hercules, CA, USA) by ramping the voltage to a maximum of 5000 V and terminated when a total of 90,000 Vh was reached. The maximum current was 50 µA per strip. After IEF, the IPG strips were reduced with DTT in the equilibration buffer (6 M urea, 0.5 M Tris/HCl, pH 6.8, 2% *w*/*v* SDS, 30% *w*/*v* glycerol and 1% *w*/*v* DTT) for 15 min and then alkylated with iodoacetamide (2.5% *w*/*v*) for an additional 20 min. After the equilibration step, the IPG strips were transferred onto 12% polyacrylamide gels (20 × 25 cm) and the second dimension was performed at 100 V for 17 h at 15 °C using a Protean Plus™ Dodeca Cell™ electrophoretic chamber (Bio-Rad, Hercules, CA, USA). Finally, Coomassie staining of gels was carried out as described previously by Pink et al. [23].

### 2.3. Image Acquisition and Data Analysis

Gels were digitally scanned using a GS-800™ Calibrated Densitometer (Bio-Rad). The 2-D images were analyzed using PDQuest Analysis software version 8.0.1 Advanced (Bio-Rad, Hercules, CA, USA). The spots present on at least six gels from each group were designated as expressed protein spots and were further analyzed. The gel that displayed the highest number of spots was marked as the master gel for matching the remaining gels. The spot volume was used as the parameter for quantifying protein expression after normalization based on the local regression model. This normalization method is less susceptible to outliers than a simple linear regression and also corrects differences in labeling efficiency at different concentration levels in the analysed 2-D gel. It also calculates a curve in the scatter plot which minimizes the distance to all points in the plot. The curve is used to calculate the normalization factor for each spot. After normalization, the volume of each spot was averaged for two replicates of each biological sample. The level of statistical significance (a *p* value less than 0.05) was first used to select differential protein spots that then were ranked by a fold-change with a cutoff of 2. The degree of difference between protein groups was expressed as an average ratio. To measure the variability within groups, the coefficient of variation (CV) was calculated for each replicate group. Based on the standard 2-D markers, the experimental isoelectric point (pI) and molecular weight (kDa) values were computed for each identified protein spot.

### 2.4. Mass Spectrometry and Bioinformatic Data Analysis

Reproducible protein spots (comprising a 2-D map) including these showing statistically significant alterations in response to CR diet were cut out from the gels and were processed as previously described by Ożgo et al. [24]. Here, we utilized two different MALDI-TOF MS instruments to identify the protein spots, namely the Microflex™ MALDI-TOF mass spectrometer (Bruker Daltonics, Bremen, Germany) and the 7T solariX™ MALDI FT-ICR mass spectrometer (SolariX 2xR, Bruker Daltonics, Bremen, Germany). 

The Microflex™ MALDI-TOF mass spectrometer was operated in a positive ion reflector mode. The mass spectra were acquired with 150 shots of a nitrogen laser operating at 20 Hz. The external calibration was performed using Peptide Mass Standard II (Bruker Daltonics) based on the monoisotopic values of the following peptides: bradykinin 1–7 (757.3992 Da), angiotensin II (1046.5418 Da), angiotensin I (1296.6848 Da), substance P (1347.7354 Da), bombesin (1619.8223 Da), ACTH clip 1–17 (2093.0862 Da), ACTH clip 18–39 (2465.1983 Da) and somatostatin 28 (3147.4710 Da), whereas an internal calibration was based on the tryptic autolytic products (842.51 and 2211.10 *m*/*z*).

Spots that were not successfully identified with the Microflex™ MALDI-TOF mass spectrometer were further subjected to analysis using the 7T SolariX 2xR MALDI FT-ICR mass spectrometer equipped with dual ESI-MALDI source and smartbeam™ II 1 kHz, 355 nm solid state Nd:YAG laser focused to a diameter of ~25 μm. The mass spectra were collected in the positive mode with 1000 laser shots from each spot. Internal mass calibration was performed using a lock mass calibration on a known *m*/*z* and external Sodium Formic Acid cluster (NaFA) calibrant. Data were acquired with the use of ftControl and analysed with the aid of the Data Analysis software (Bruker Daltonics, Bremen, Germany).

All peptide mass fingerprinting (PMF) data were compared to vertebrate databases (SWISS-PROT; http://us.expasy.org/uniprot/ (accessed on 19 October 2020) and NCBI; http://www.ncbi.nlm.nih.gov/ (accessed on 19 October 2020) with the aid of MASCOT search engine (http://www.matrixscience.com/) in Protein Scape 4.2 software (Bruker Daltonics, Bremen, Germany). Search criteria included: trypsin as an enzyme, carbamidomethylation as a fixed modification; oxidation (M) as a variable modification was set for the Microflex™ MALDI-TOF MS data, and deamidation (NQ), oxidation (M) and Gln→pyro-Glu (N- term Q) were set as variable modifications for the SolariX 2xR MRMS data. Peptide mass tolerance was from 50 to 150 ppm and a maximum of two missed cleavage sites. Contaminating peaks of keratin and trypsin were removed manually from the peptide mass list prior to database search. The results of PMF-based identification were accepted when the protein score was significant (*p* < 0.05) with at least 9 matching peptides and 15% sequence coverage. 

Gene Ontology (GO) enrichment analysis and protein–protein interactions (PPI) of all 62 identified cecal proteins were performed with STRING v11 [25], using porcine orthologs and the functional database. The web-based Search Tool for the Retrieval of Interacting Genes/Proteins (STRING) database was used with the following options selected: interaction score of 0.400 (medium confidence) and identification of significant results based on a Benjamini–Hochberg False Discovery Rate (FDR)-adjusted *p*-value < 0.05. Functional association was made from the STRING cluster enrichment tables. 

### 2.5. Statistical Analysis

Statistical analyses were performed using the IBM SPSS package (IBM Corp.; SPSS Statistics for Windows; Version 23.0, Armonk, NY, USA) to detect differences in protein expression level in response to CR diet and individual pig was used as the experimental unit. Normality was checked using the Shapiro–Wilk test that showed that some data were not normally distributed. Production traits and spots, which showed a normal distribution, were further analysed using Student’s *t*-test, whereas non-parametric Mann–Whitney tests were applied to not normally distributed protein spots, and *p* < 0.05 was considered as significant. 

## 3. Results

### 3.1. Production Traits

All piglets remained healthy throughout the whole experimental period. No significant differences between the two groups related to body weight (BW) gain and final BW were observed. A similar trend was also found for feed intake, but it could not be analysed statistically as it was measured only per pen. The above-mentioned data were previously described [17] and can also be found in Table 3.

### 3.2. Two-Dimensional (2-D) Gel-Based Cecal Mucosa Protein Profile of 50-Day-Old Piglets

**Figure 1:**
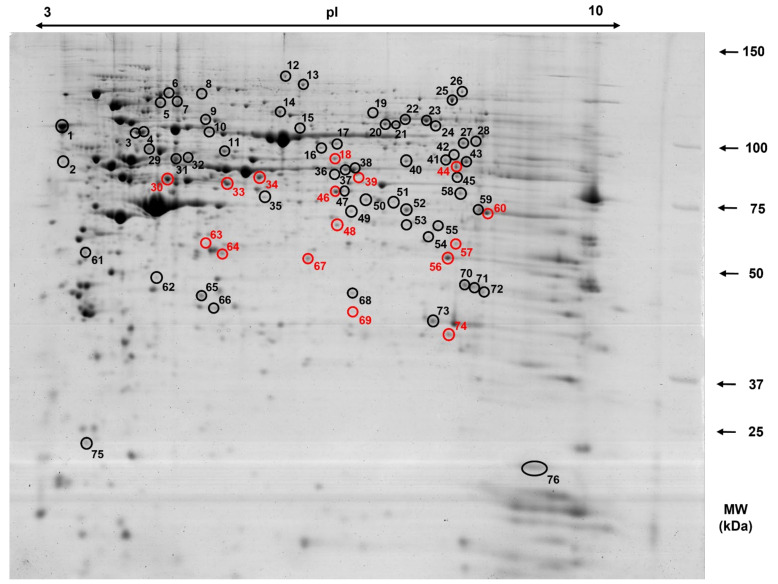
Representative 2-D gel image of cecal mucosa protein profile of 50-day-old piglets. Samples of 1000 µg of protein were applied onto the IPG strip (24 cm, pH 3–10) for the first dimension; the second dimension was performed on 12% SDS-PAGE gels that were subsequently stained with Coomassie brilliant blue G-250. Numbered spots were identified using MALDI-TOF and MALDI FT-ICR MS after in-gel digestion of proteins by trypsin. The red circles indicate protein spots that were statistically altered in the cecal mucosa of nursery pigs fed a diet enriched with 4% of dried chicory root. The black circles along with the red ones indicate all identified spots that comprise 2-DE-based protein map of porcine cecal mucosa. Spot numbers correspond to those in Table 4 and Table 5.

In order to establish a 2-D map of proteins expressed in cecal mucosa of piglets we utilized 2-DE coupled with MALDI- TOF and MALDI FT-ICR mass spectrometry. A comparative bioinformatic analysis of eight different cecal mucosa protein extracts collected from pigs fed a control (C) diet revealed the presence of 490 ± 51 protein spots per each analysed 2-D gel with molecular masses ranging from 25 to 150 kDa and pI values of 3–10. A representative gel image of expressed proteins in the cecal mucosa of 50-day-old PIC × Penarlan P76 piglets is shown in Figure 1, in which all identified spots are marked with numbered circles. Protein spots with similar locations and expression variance on each gel (350) were selected for the MS analysis. These spots constituted 65% of all detected protein features. The average CV in normalized spot volume was determined to be 56.41%. All reproducible protein spots (350) were excised from the gels and subjected to peptide mass fingerprinting using MALDI-TOF and MALDI FT-ICR MS. In total, 76 spots were successfully identified which corresponded to 62 different gene products. A high proportion of these protein spots (74%) were identified based on *Sus scrofa* identities. A minimum Mascot score of 62 and matched mass values of 5 were used to identify proteins. Proteins were identified with a sequence coverage from 15 to 77%. A comprehensive list of all identified proteins, number of peptides matched, sequence coverage, Mascot scores, experimental and theoretical pI values and molecular weights along with the taxonomy are given in Table 4.

The 2-D gel analysis showed that nine proteins were expressed as multiple spots, indicating that they were isoforms, whereas the remaining fifty-three proteins were resolved as single ones. Elongation factor 1-gamma (spots no. 13, 37, 46, 47), adenylyl cyclase-associated protein 1 (spots no. 40, 44, 45) and gelsolin (spots no. 3, 4, 12) were expressed as a multiple spots, as these proteins were detected by 2-DE as four, three and three proteoforms, respectively, whereas calreticulin precursor (spots no. 1, 2), moesin isoform X1 (spots no. 20, 21), transferrin (spots no. 22, 23), tubulin alpha-1B chain (spots no. 31, 32), UDP-glucose 6-dehydrogenase (spots no. 42, 43) and thiosulfate sulfurtransferase isoform X2 (spots no. 70, 71) were represented on the 2-D gels by two spots. For each identified protein spot, we compared the theoretical coordinates of pI and MW accessible in the Uniprot or NCBI databases with the experimental values of these parameters using the PDQuest 8.01 advanced pI/Mw tool (Table 4). The majority of the computed and theoretical pI coordinates were consistent with a minimal variation. Nevertheless, almost all protein spots showed the localization shift to the larger masses zones. The 2-DE analysis of the cecal mucosa proteins revealed a computed pI range from 3.70 (calreticulin precursor, spots no. 1 and 2) to 9.20 (cofilin-1, spot no. 76). An acidic pI (68% of the identified spots) was reported to be predominant over a basic pI (32%). The spectrum of estimated molecular mass of the cecal proteins varied from 20.20 (cofilin-1, spot no. 76) to 134.80 kDa (gelsolin, spot no. 12). It should be noted that the majority (97%) of the identified proteins were high-molecular-weight proteins (>30 kDa).

### 3.3. Differentially Expressed Proteins in the Cecal Mucosa in Response to CR Diet

The CR diet was shown to induce significant changes in the expression of 16 cecal mucosa proteins. Among them, 15 proteins were down-regulated, while only one was shown to be up-regulated when compared to the control (C) group. The analysis of intragroup variation showed that CV for replicate groups were 56.41% and 50.64% for group C and CR, respectively. A representative gel image is shown in Figure 1, in which all significantly altered proteins in response to CR diet are marked with the red circles. A comprehensive list of all differentially expressed proteins along with the average ratio of expression for protein spots found in the cecum of pigs fed the CR diet compared with the C diet are given in Table 5.

### 3.4. Functional Annotations and Pathway Enrichment Analysis of Porcine Cecal Mucosa Proteins 

**Figure 2:**
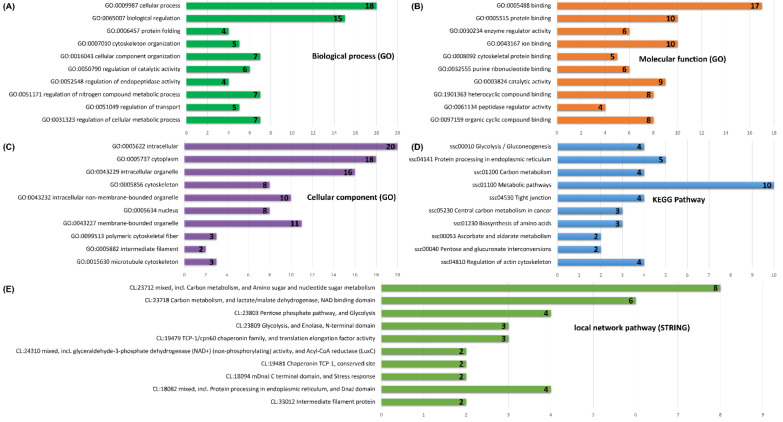
The top ten (the most statistically significant according to Benjamini–Hochberg FDR) predicted enrichment function and pathway terms of porcine cecal proteins retrieved from the STRING database: (**A**) Classification of proteins based on Gene Ontology (GO) biological process; (**B**) classification of proteins based on GO molecular function; (**C**) classification of proteins based on GO cellular component; (**D**) pathway analysis of proteins assessed by the KEGG classification; (**E**) pathway analysis of proteins identified by the local network cluster (STRING) classification.

For functional annotation of Gene Ontology (GO) and analysis of pathway enrichment of all identified cecal proteins, we used the web-based STRING v11 tool utilizing porcine orthologs and their functional database [25]. Selecting as input the list of reproducible protein spots (comprising a 2-D map) and including those showing statistically significant alterations in response to the CR diet (Table 4), we performed GO enrichment analysis. The biological functions were defined from three aspects: biological process, molecular function and cellular component. Statistical analysis parameters within STRING v11 (Benjamini–Hochberg False Discovery Rate (FDR)-adjusted *p*-value < 0.05) allowed us to include between thirty-two and eighty-three proteins per category (Supplementary Tables S1–S3). In the biological process category, the most significant terms were related to cellular process (*p*-value = 6.55 × 10^−9^), biological regulation (*p*-value = 1.06 × 10^−7^), protein folding (*p*-value = 1.13 × 10^−5^) and cytoskeleton organization (*p*-value = 1.06 × 10^−7^) (Supplementary Table S1, Figure 2A). The most represented terms in the molecular function group were proteins binding with other proteins (*p*-value = 4.52 × 10^−6^), ions (*p*-value = 6.04 × 10^−6^), cytoskeleton (*p*-value = 1.20 × 10^−5^), purine ribonucleotides (*p*-value = 2.80 × 10^−5^) as well as those displaying enzyme regulator (*p*-value = 6.04 × 10^−6^) and catalytic activities (*p*-value = 3.14 × 10^−5^) (Supplementary Table S2, Figure 2B). Finally, the most overrepresented GO terms in the cellular component category showed that these proteins are located in various areas within the cell, including cytoplasm (*p*-value = 8.45 × 10^−12^), intracellular organelle (*p*-value = 2.59 × 10^−10^), cytoskeleton (*p*-value = 3.71 × 10^−9^) and nucleus (*p*-value = 5.35 × 10^−6^) (Supplementary Table S3, Figure 2C).

To elucidate molecular mechanisms underlying the functioning of the retrieved cecal proteins, we performed KEGG pathway and local network pathway (STRING) enrichment analysis. KEGG pathway ranking by statistical enrichment showed the involvement of porcine cecal proteins in the process of glycolysis and gluconeogenesis (*p*-value = 0.0013), protein processing in endoplasmic reticulum (*p*-value = 0.0016), carbon metabolism (*p*-value = 0.0042), biosynthesis of amino acids (*p*-value = 0.0071) and tight junctions (*p*-value = 0.0066) (Supplementary Table S4, Figure 2D). The local network pathway (STRING) results tie in well with those obtained from the KEGG pathway enrichment system (Supplementary Table S5). Figure 2E shows that cecal proteins are mainly associated with carbon metabolism (*p*-value = 2.54 × 10^−6^), pentose phosphate pathway and glycolysis (*p*-value = 0.0001), as well as protein processing in the endoplasmic reticulum (*p*-value = 0.0122).

**Figure 3:**
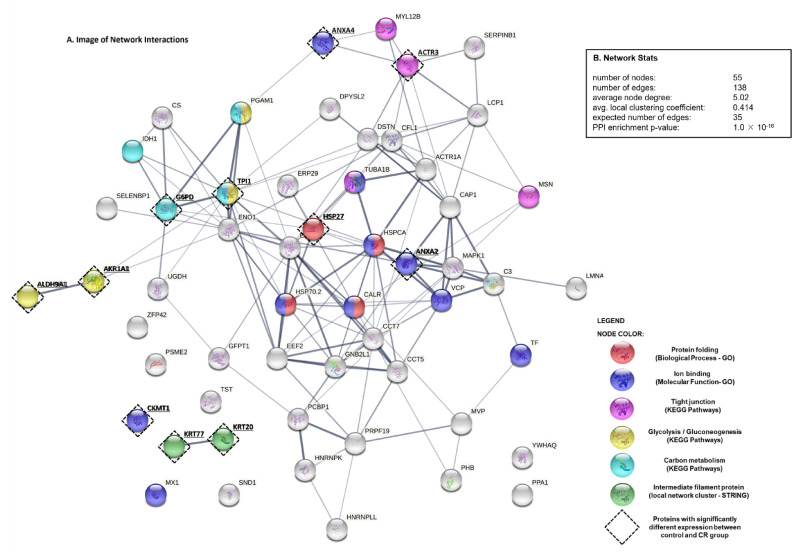
STRING analysis of proteins identified by MALDI-TOF MS and MALDI FT-ICR MS: (**A**) Protein–protein interaction image displaying nodes and edges between all identified proteins found in the cecal mucosa of nursery pigs. Proteins are represented as nodes. Various thickness of the edges indicates the strength and nature of the presented interactions detected between the proteins. Different colours of nodes shows selected gene ontology (GO) terms such as biological process, molecular function, KEGG pathways and local network cluster (STRING) that are associated with the porcine cecal proteins. Black dotted edgings along selected nodes represent cecal proteins that were found to be significantly altered in the group of pigs fed a diet enriched with 4% dried chicory root. Full name of identified proteins along with the corresponding genes can be found in Table 4 and Table 5; (**B**) network statistics presenting data concerning included number of nodes and edges, the average node degree, the average local clustering coefficient, the expected number of edges, and the protein–protein interaction (PPI) enrichment *p*-value.

The protein–protein interactions (PPI) network analysis using the STRING v11 tool (Figure 3) demonstrated a statistically significant association among the top 55 proteins (PPI enrichment *p*-value = 1.0 × 10^−16^). The PPI network was constructed using proteins (as nodes) and their known and predicted interactions (as edges). It was shown that cecal proteins display more interactions among themselves (138 edges) compared to predicted interactions based on the structural, genomic and biological context of proteins (expected number of edges: 38), indicating that these proteins are to some extent functionally connected. We also utilized this PPI network as a background to highlight predicted interactions that may exist between differentially expressed proteins in the group of animals fed the CR diet. In that manner, we used colored nodes that represent selected GO terms such as biological process (protein folding, *p*-value = 1.13 × 10^−5^), molecular function (ion binding, *p*-value = 6.04 × 10^−6^), KEGG pathways (tight junctions, *p*-value = 0.0066; glycolysis/gluconeogenesis, *p*-value = 0.0013; carbon metabolism, *p*-value = 0.0042) and local network cluster (STRING) (intermediate filament protein, *p*-value = 0.0142). 

### 3.5. STRING Analysis of Protein Networks

To seek potential interactions between all identified porcine cecal proteins, the STRING tool was employed. The protein–protein interaction (PPI) network for cecal mucosa proteins was constructed and used as a background to better understand the interactions of proteins that were found to be statistically altered in response to CR diet. As shown in Figure 3, selected proteins that were changed by a CR diet (marked with black circles) are coloured according to the most significant—GO: biological process or GO: molecular function—and signaling pathways. 

## 4. Discussion

### 4.1. 2-D Gel-Based Cecal Mucosa Protein Profile of 50-Day-Old Piglets

To the best of our knowledge, this article presents the first 2-DE-based protein map of the porcine cecal mucosa. The data obtained in the current study are to some extent consistent with and complementary to the findings of two recent works on proteomic characterisation of mucus samples from the entire porcine GIT [21,26]. In comparison to these large-scale studies, only 62 differentially expressed proteins (76 spots) were identified on 2-D gels in the present work. This result is significantly lower than the 3576 proteins detected by Barmpatsalou et al. [21] in the cecum of 20–22-week-old pigs using a label-free and targeted mass tandem (TMT-labelled) proteomic approach. This was due to technical obstacles associated with gel-based techniques that arose from physical separation constraints, such as protein size, extreme pI range and protein hydrophobicity. Although 2-DE cannot visualize the complete proteome, its main advantage is that post-translational modifications can be observed and protein experimental isoelectric point and molecular mass can be obtained. In our study, among the successfully identified protein spots, nine were expressed as multiple spots, indicating that they were isoforms. These proteoforms showed slight changes in molecular weight and pI due to various naturally occurring protein post-translational modifications or proteolytic protein processing (precursor vs. mature protein form) [27]. Most of the computed and theoretical pI coordinates were consistent and showed minimal variation. Nevertheless, almost all protein spots showed a shift in localisation to zones with higher masses. This was probably due to protein heterogeneity associated with glycosylation, oligomerisation of proteoforms, or covalent linkage with protein partners [27]. 

Homeostasis of the gut barrier is highly dependent on interactions between several components. These include the adhesive gel mucus layer, epithelial cells and intracellular tight junctions, as well as the underlying lamina propria [28]. The cecal mucus that covers and protects the single layer of epithelial cells is also believed to be a dynamic system coupled to the immune system via goblet cells that are involved in immune surveillance and directing the host response [29]. The large intestinal mucus gel network is not solely composed of mucins, predominantly mucin-2, which provides structural and functional framework, but it is also rich in extracellular/plasma proteins secreted into the mucus from both goblet cells and enterocytes [30]. A recent study by Barmpatsalou et al. [21] provided fundamental knowledge about the physiological properties, composition and structural profiling of porcine mucus from the entire GIT. The results clearly show that the large and small intestine, given their anatomical and functional differences, exhibit distinctive protein expression patterns. As a consequence of its central role in food processing and nutrient extraction, jejunal mucus was found to contain high levels of proteins associated with digestion and absorption, whereas antimicrobial peptides and proteins involved in the immune system pathways were mainly observed in the colonic mucosa. These authors also observed the up-regulation of proteins associated with the metabolism of lipids, proteins, carbohydrates and amino acids in the entire porcine GIT mucosa [21]. Our findings are in line with the aforementioned study, as detailed annotations of the GO-biological process, KEGG pathway and local network pathway (STRING) clearly indicate that metabolic pathways were overrepresented in the porcine caecal mucosa under normal physiological conditions. Based on GO enrichment analysis, we have established that the main functions of the identified proteins were carbohydrate metabolism, including glycolysis and gluconeogenesis processes, carbon metabolism and amino acid synthesis, which is consistent with physiological functions as well as with gut microbiota communities inhabiting the cecum [26,31,32]. In line with previous studies [26], we also demonstrated the presence of several tight junction-associated proteins (TUBA1B, MYL12B, MSN, ACTR3), as well as those involved in the regulation of the actin cytoskeleton (MYL12B, MAPK1, MSN, CFL1). Accumulating evidence from basic and clinical studies suggests that both the actin cytoskeleton and the molecular components of tight junctions are essential for maintaining epithelial integrity and barrier function [33].

### 4.2. Differentially Expressed Proteins in the Cecal Mucosa in Response to CR Diet

Our subsequent research efforts also sought to determine the effect of a diet supplemented with 4% dried chicory root on the protein profile of the cecal mucosa in pigs. A comparative analysis of cecal protein expression patterns enabled us to identify 16 differentially accumulated protein spots between the control group and the CR group. The cecal PPI network analysis showed that its biological processes and pathways could be implicated in metabolism and its regulation, cytoskeleton organization, tight junction protein assembly, and ion binding. We found that 40 days of supplementation with the CR diet caused a significant decrease in the expression of metabolism-associated proteins, including enzymes involved in glycolysis (G6PD, TPI1, ALDH9A1, CKMT1 and AKR1A1). These changes in glycolytic protein expression were mainly associated with the direct effect of specific, energy-providing, substrate preferences and were consistent with our previous studies in pigs fed diets supplemented either with 1% or 3% native chicory inulin [20]. Although cecal tissue can utilise different energy-rich substrates, such as glucose and glutamine, butyrate is preferred as a primary energy source. In our opinion, the trend of glycolytic enzyme expression can be explained by the increased production of SCFAs in the large intestine of pigs following dietary chicory root treatment. Chicory is a rich source of dietary fibres, such as inulin and FOS, which have been found to be fermented by gut commensal bacteria, and thus generate SCFAs, mainly acetate, propionate and butyrate [7]. It should be noted that the concentration of these acids varies across different segments of the GI tract. According to Diao et al. [34], significantly lower levels of SCFAs were detected in the ileum (15 μmol/g digesta), while higher concentrations were recorded in the cecum (110 μmol/g of digesta) and colon (102 μmol/g digesta) in weaned piglets. It has been estimated that about 95% of SCFAs produced are rapidly taken up by intestinal epithelial cells, while the remaining 5% are excreted in the feces [7]. There are two primary mechanisms of apical uptake of SCFAs by intestinal epithelial cells. They can be transported either by a non-ionic diffusion of protonated acids and/or via an active transport, where dissociated SCFA forms require specific transport proteins to facilitate their passage across the cell membrane. Active transport of SCFAs from the lumen to the cytosol of intestinal epithelial cells involves monocarboxylate transporter 1 (MCT1) and sodium monocarboxylate transporter 1 (SMCT1) [7,35]. Once absorbed, SCFAs serve as an energy source. Among the three main SCFAs, butyric acid exerts the most beneficial effect in this respect, as its metabolism accounts for 80% of colonocyte energy requirements [36]. This result is consistent with a previous study reporting butyrogenic and bifidogenic effects of chicory forage administration in pigs [37]. The decreased expression of glycolytic enzymes found in the current study indicate increased amounts of butyrate, i.e., the preferred energy source of the cecal epithelium.

We also found that the expression level of four structural proteins was significantly altered, including KRT20, which showed a more than two-fold increase in the CR group. Keratins are a group of intermediate filament proteins responsible for mechanical support of epithelial cells. Moreover, these proteins also regulate cell proliferation, migration and can exert a protective effect against oxidative stress [38]. KRT20, one of the major type I keratins, has also been associated with mucin secretion and actin filament organisation [39] and, above all, is a well-established intestinal differentiation marker [40]. As reviewed by Polari et al. [38], animal studies have provided solid evidence that this protein is expressed at low levels in the bottom of the colon crypt, but its expression increases substantially in the differentiated luminal cells. The results obtained in our study suggest that SCFAs, and particularly butyrate, enhanced KRT20 expression in the group of animals fed a diet supplemented with chicory root, indicating its vital function in the differentiation of caecal epithelial cells. These findings are consistent with an earlier study showing that incubation of Caco-2 cells in medium with β hydroxybutyrate could significantly increase KRT20 expression [41]. Our previous study performed in the same group of animals showed an increase in the abundance of ileal KRT20 and KRT8 proteins in pigs fed a diet enriched with chicory root [8]. On the other hand, a work by Kien et al. [42] provided strong evidence that when butyrate production was abnormally increased by feeding a fermentable carbohydrate, cecal cell proliferation was reduced. The results of the current study seem to support the aforementioned findings, as they demonstrate down-regulation of two proteins involved in molecular mechanisms controlling protein biosynthesis (PRPF19, EEF1G), as well as those belonging to the group of structural proteins (ACTR3, KRT77, CAP1 and actin). These results differ from our earlier study, where a diet supplemented with 3% inulin caused a pronounced increase in the expression of these proteins, particularly in the caecal and distal colonic mucosa of nursery pigs [20]; however, they are largely consistent with our other studies, in which we reported a similar pattern of changes for ileal proteins, but only in pigs fed a diet supplemented with 1% native chicory inulin [43]. Unfortunately, on the basis of the results obtained, we cannot explain the exact mechanism of the observed contradictory effects. Nevertheless, taken together, these findings may indicate a reduced cecal cell turnover rate in response to the CR diet. 

Another interesting finding is that the CR diet caused a more than three-fold decrease in the expression of leucocyte elastase inhibitor (SERPINB1) in the cecal mucosa of pigs. A related animal study has suggested that SERPINB1 was involved in intestinal barrier dysfunction by disrupting tight junction, and thus could play an important role in the pathogenesis of inflammatory bowel disease [44]. We also found that the experimental diet significantly down-regulated the expression of both ANXA4 (two-fold) and ANAXA2 (three-fold), which belong to the family of calcium-regulated phospholipid-binding proteins, as well as samll heat shock protein, namely, HSP27 (3-fold) whose higher abundance in colonic tissue was associated with increased proliferation, migration and invasion of colon cancer cells [45,46]. This observation may be explained by the fact that chicory root contains a fairly large amount of phytometabolites, such as guaianolides, 6-methoxyflavone, eudesmanolides, germacranolides, polyacetylene, sterol, anthocyanin, delphinidin and 3,4-dihydroxyphenethyl, which show anticancer activity [47].

## 5. Conclusions

In summary, we have established a 2-DE map of the cecal mucosa proteome in 50-day-old piglets and identified several differentially expressed proteins using MALDI-TOF and MALDI FT-ICR mass spectrometry. Unfortunately, an identity could only be assigned to 22% (76 spots) of the total of 350 reproducible spots detected on 2-D gels. Nevertheless, given the age-mediated changes in the physiology and function of the gut, our results may complement existing data and provide a basis for further proteomic research in this area.

Contrary to expectations, we found that the CR diet caused down-regulation of proteins engaged in transcriptional and translational activity, as well as several structural proteins, which could indicate a decreased caecal cell turnover rate. On the other hand, the altered abundance of several cecal proteins, such as KRT20, SERPINB1, ANAXA2 and ANAXA4, could indicate positive effects of the CR diet on the gut health of nursery pigs. Several questions remain unanswered, and thus additional studies are required to obtain a complete picture of the molecular mechanism behind the effects of prebiotics on the cecal mucosa.

As a result of technical hurdles associated with 2-DE due to physical separation constraints such as protein size, extreme pI range or protein hydrophobicity, our results can be considered preliminary and require further validation using more sophisticated proteomic techniques. Therefore, our research will be expanded to include alternative approaches, such as the promising gel-free proteomics approach.

## Figures and Tables

**Table 1:** Composition of the control diet (C) and the diet supplemented with 4% dried chicory root (CR).

Diet	C Group	CR Group
Composition (g/kg)		
Wheat	468.40	458.40
Barley	200.00	200.00
Corn starch	30.00	0.00
Full-fat soy bean	59.00	59.00
Whey	97.00	97.00
Fish meal	40.00	40.00
Spray-dried blood plasma	40.00	40.00
Soybean oil	34.00	34.00
Calcium formate	3.00	3.00
Calcium carbonate	5.00	5.00
Calcium monophosphate	6.00	6.00
Sodium chloride	0.70	0.70
L-Lysine HCL (78.5%)	6.10	6.10
DL-Methionine (99%)	2.30	2.30
L-Threonine (98%)	2.60	2.60
L-Tryptophan (98%)	0.90	0.90
Mineral–vitamin mix ^1^	4.00	4.00
Aroma	1.00	1.00
Dried chicory roots	0.00	40.00
Dry mater (%) and nutrient (% dry matter) ^2^		
Dry matter	90.03	90.15
Crude ash	4.54	4.55
Crude protein	20.05	20.05
Ether extract	6.04	6.04
Crude fibre	1.52	1.53
Fructans	1.00	2.74
ME (MJ/kg) ^3^	14.30	14.20

^1^ Mineral-vitamin mix provided per kg of a diet: vitamin A 2400 IU, vitamin D3 240 IU, vitamin E 12 mg, vitamin K3 480 µg, vitamin B1 480 µg, vitamin B2 960 µg, vitamin B6 960 µg, nicotinic acid 6.4 mg, pantothenic acid 3.2 mg, folic acid 640 µg, biotin 40 µg, vitamin B12 6.4 µg, choline chloride 48 mg, Mg 3.2 mg, Fe 24 mg, Zn 22.4 mg, Mn 9.6 mg, Cu 25.6 mg, I 0.16 mg, Se 64 µg, Co 64 µg. ^2^ Analyzed values. ^3^ Metabolizable energy, calculated values.

**Table 2:** Chemical composition (%) of dried chicory root (CR).

Item	CR ^1^
Dry matter	92.67
Crude protein	5.63
Crude ash	3.61
Ether extract	0.34
Crude fiber	5.56
Fructans	51.56

^1^ Dried root of *Cichorium intybus* L. produced by VITAMIX, Niemcz, Poland.

**Table 3:** Feed intake and growth parameters of piglets fed the control diet (C) and the diet supplemented with 4% of dried chicory root (CR).

Parameter	C	CR ^1^	SEM	*p*-Value
Initial body weight (at weaning), kg	8.00	8.60	0.26	0.256
Average daily feed intake, kg/day	0.53	0.78	0.075	-
Final body weight, kg	17.9	19.1	0.72	0.427
Total body weight gain, kg	9.9	10.5	0.58	0.628
Average daily body weight gain, kg/day	0.47	0.50	0.028	0.624

^1^ Dried root of *Cichorium intybus* L. produced by VITAMIX, Niemcz, Poland. Data are presented as means and pooled SEM and were calculated for the period from 28th (weaning) to 50th day of a piglet’s life. Feed intake was not analysed statistically because it was only estimated on a per-pen basis.

**Table 4:** List of protein spots identified by MALDI-TOF MS and MALDI FT-ICR MS found in the cecal mucosa of nursery pigs. Spot numbers correspond to those in Figure 1.

Spot No.	Gene Name ^3^	MVM ^4^/ MS ^5^	SC ^6^	Theoretical pI/Mw (pH/kDa) ^7^	Experimental pI/Mw (pH/kDa) ^8^	Taxonomy
1	CALR ^1^	8/92	34	4.32/48.43	3.70/107.60	*Sus scrofa*
2	CALR ^1^	8/84	30	4.32/48.43	3.70/91.90	*Sus scrofa*
3	GSN ^1^	6/67	15	5.93/85.06	4.60/105.00	*Sus scrofa*
4	GSN ^1^	6/67	15	5.93/85.06	4.70/105.40	*Sus scrofa*
5	LCP1 ^1^	10/81	24	5.29/70.65	4.80/119.80	*Sus scrofa*
6	MX1 ^1^	6/89	15	5.45/76.27	4.90/124.10	*Sus scrofa*
7	ARHGDIA ^1^	8/97	40	5.12/23.44	5.10/119.50	*Sus scrofa*
8	MVP ^1^	17/137	31	5.53/99.98	5.30/124.40	*Sus scrofa*
9	HSPCA ^1^	11/105	23	4.93/85.12	5.40/111.30	*Sus scrofa*
10	HSP70.2 ^1^	10/106	26	5.60/70.43	5.50/105.30	*Sus scrofa*
11	CCT5 ^1^	7/88	29	5.22/53.64	5.70/96.50	*Pan troglodytes*
12	GSN ^1^	14/159	31	5.93/85.06	6.30/134.80	*Sus scrofa*
13	EEF1G ^1^	7/90	31	6.16/49.93	6.60/129.20	*Sus scrofa*
14	YWHAQ ^1^	10/112	65	4.37/18.36	6.30/114.80	*Sus scrofa*
15	HSP8 ^1^	8/80	24	5.38/71.10	6.60/107.30	*Chrysochloris asiatica*
16	SARS1 ^1^	7/65	22	5.86/59.06	6.80/98.00	*Rattus norvegicus*
17	DPYSL2 ^1^	9/78	27	5.95/62.64	6.90/100.30	*Bos taurus*
18	PRPF19 ^1^	8/82	26	6.14/55.60	7.00/93.30	*Sus scrofa*
19	GFPT1 ^1^	8/69	16	6.39/79.29	7.40/114.80	*Mus musculus*
20	MSN ^1^	34/259	44	6.18/68.02	7.50/108.90	*Sus scrofa*
21	MSN ^1^	15/131	28	6.18/68.02	7.60/108.60	*Sus scrofa*
22	TF ^1^	9/80	20	6.73/78.95	7.70/111.60	*Sus scrofa*
23	TF ^1^	10/70	20	6.93/78.97	8.20/111.30	*Sus scrofa*
24	LMNA ^1^	28/241	49	6.40/65.19	8.20/107.50	*Sus scrofa*
25	EEF2 ^1^	43/396	56	6.41/96.26	8.30/120.70	*Sus scrofa*
26	SND1 ^1^	23/201	28	6.72/102.52	8.40/125.30	*Sus scrofa*
27	HNRNPLL ^1^	15/135	26	8.46/64,57	8.50/100.10	*Sus scrofa*
28	C3 ^1^	31/208	24	5.99/193.05	8.60/101.50	*Sus scrofa*
29	HNRNPK ^1^	9/76	28	5.39/51.23	4.70/97.00	*Sus scrofa*
30	KRT20 ^1^	16/176	52	5.38/49.09	4.90/85.20	*Sus scrofa*
31	TUBA1B ^1^	9/93	35	4.94/50.80	5.00/92.50	*Sus scrofa*
32	TUBA1B ^1^	10/85	33	4.94/50.87	5.20/92.20	*Rousettus aegyptiacus*
33	ACTR3 ^1^	19/130	57	5.61/47.80	5.60/83.00	*Bos taurus*
34	ALDH9A1 ^1^	12/62	29	5.84/54.82	5.70/83.00	*Sus scrofa*
35	VCP ^1^	14/127	31	5.13/89.92	6.10/78.10	*Sus scrofa*
36	ENO1 ^1^	11/80	28	6.44/47.60	7.00/86.80	*Sus scrofa*
37	EEF1G ^1^	10/130	38	6.16/49.93	7.20/88.50	*Sus scrofa*
38	SELENBP1 ^1^	18/161	41	5.93/52.93	7.20/88.50	*Homo sapiens*
39	G6PD ^1^	8/93	29	6.27/59.71	7.30/86.30	*Sus scrofa*
40	CAP1 ^1^	8/62	23	7.64/51.88	7.70/92.20	*Macaca fascicularis*
41	CCT7 ^1^	11/64	26	6.78/59.92	8.30/92.20	*Bos taurus*
42	UDPGDH1 ^1^	16/124	42	6.73/55.72	8.30/95.00	*Sus scrofa*
43	UDPGDH1 ^1^	14/109	39	6.73/55.72	8.50/92.00	*Sus scrofa*
44	CAP1 ^1^	11/109	27	6.84/51.56	8.30/89.30	*Sus scrofa*
45	CAP1 ^1^	8/95	24	7.62/51.77	8.30/86.00	*Sus scrofa*
46	EEF1G ^1^	17/178	52	6.16/49.93	7.00/80.40	*Sus scrofa*
47	EEF1G ^1^	17/145	56	6.15/50.38	7.10/74.50	*Sus scrofa*
48	SERPINB1 ^1^	14/142	43	6.13/42.66	7.00/66.20	*Sus scrofa*
49	ACTR1A ^1^	5/64	30	6.19/42.70	7.30/77.10	*Canis lupus familiaris*
50	SEPTIN2 ^1^	7/64	34	6.15/41.69	7.30/77.10	*Homo sapiens*
51	ZFP42 ^1^	8/75	31	9.12/35.52	7.70/76.40	*Homo sapiens*
52	IDH1 ^1^	14/140	48	6.48/47.15	7.80/73.00	*Sus scrofa*
53	MAPK1 ^1^	12/169	42	6.50/41.68	7.80/65.90	*Sus scrofa*
54	AKR1A1 ^1^	9/91	39	6.51/36.84	8.10/61.40	*Sus scrofa*
55	PCBP1 ^1^	16/184	60	6.66/37.99	8.20/66.50	*Sus scrofa*
56	ANXA2 ^2^	39/81	77	6.49/38.79	8.30/53.60	*Sus scrofa*
57	AKR1A1 ^2^	26/75	64	6.51/36.84	8.30/58.80	*Sus scrofa*
58	6PGD ^1^	24/207	50	6.48/53.66	8.40/79.50	*Sus scrofa*
59	CS ^1^	17/126	38	6.92/48.90	8.60/74.20	*Sus scrofa*
60	CKMT1 ^1^	20/222	47	8.60/47.30	8.70/71.60	*Sus scrofa*
61	IPP1 ^1^	7/90	43	5.44/33.25	4.00/55.50	*Sus scrofa*
62	ATP5F1B ^1^	10/87	28	5.15/56.25	4.80/48.80	*Bos taurus*
63	ACT ^2^	18/50	66	5.12/42.52	5.40/58.80	*Molgula oculata*
64	ANXA4 ^2^	33/71	76	5.10/48.40	5.60/55.30	*Sus scrofa*
65	PSME2 ^1^	5/62	33	5.41/27.45	5.40/46.40	*Sus scrofa*
66	PHB ^1^	8/97	48	5.57/29.86	5.50/44.90	*Homo sapiens*
67	KRT77 ^2^	52/64	74	5.48/57.39	6.60/53.50	*Rattus norvegicus*
68	ERP29 ^1^	8/84	36	6.85/29.32	7.20/46.80	*Sus scrofa*
69	HSP27 ^2^	5/63	30	6.23/22.98	7.20/45.80	*Sus scrofa*
70	TST ^1^	16/164	56	6.72/33.65	8.50/47.70	*Sus scrofa*
71	TST ^1^	19/188	52	6.72/33.65	8.60/47.60	*Sus scrofa*
72	GNB2L1 ^1^	18/249	78	7.60/35.51	8.70/47.00	*Sus scrofa*
73	PGAM1 ^1^	13/151	68	6.67/28.90	8.10/43.30	*Homo sapiens*
74	TPI1 ^2^	11/147	58	6.54/26.88	8.30/41.70	*Sus scrofa*
75	MYL12B ^1^	7/80	50	4.66/19.85	4.00/23.80	*Echinops telfairi*
76	CFL1 ^1^	9/121	62	8.16/18.79	9.20/20.20	*Sus scrofa*

^1^ Proteins identified using the Microflex™ MALDI-TOF mass spectrometer (Bruker Daltonics, Germany). ^2^ Proteins identified using the 7T solariX™ MALDI FT-ICR mass spectrometer (SolariX 2xR, Bruker Daltonics, Germany). ^3^ Full name of proteins encoded by genes: Calreticulin precursor (CALR), Gelsolin (GSN), Plastin-2 (LCP1), Interferon-induced GTP-binding protein Mx1 (MX1), rho GDP-dissociation inhibitor alpha (ARHGDIA), Major vault protein (MVP), Heat shock protein HSP 90-alpha (HSPCA), Heat shock 70 kDa protein 1B (HSP70.2), T-complex protein 1 subunit epsilon isoform X1 (CCT5), Elongation factor 1-gamma (EEF1G), Tyrosine 3-monooxygenase/tryptophan 5-monooxygenase activation protein, theta polypeptide (YWHAQ), Heat shock cognate 71 kDa protein isoform X1 (HSP8), Serine--tRNA ligase, cytoplasmic (SARS1), Dihydropyrimidinase-related protein 2 (DPYSL2), Pre-mRNA processing factor 19 (PRPF19), Glutamine--fructose-6-phosphate aminotransferase [isomerizing] 1 (GFPT1), Moesin isoform X1 (MSN), Transferrin (TF), Prelamin-A/C (LMNA), Elongation factor 2 isoform X1 (EEF2), Staphylococcal nuclease domain-containing protein 1 (SND1), Heterogenous nuclear ribonucleoprotein L (HNRNPLL), Complement C3 isoform X1 (C3), Heterogenous nuclear ribonucleoprotein K (HNRNPK), Keratin, type I cytoskeletal 20 (KRT20), Tubulin alpha-1B chain (TUBA1B), Actin-related protein 3 (ACTR3), 4-trimethylaminobutyraldehyde dehydrogenase (ALDH9A1), Transitional endoplasmic reticulum ATPase (VCP), Alpha-enolase isoform X1 (ENO1), Selenium-binding protein 1 (SELENBP1), Glucose-6-phosphate 1-dehydrogenase isoform X2 (G6PD), Adenylyl cyclase-associated protein 1 (CAP1), T-complex protein 1 subunit eta (CCT7), UDP-glucose 6-dehydrogenase (UDPGDH1), Leukocyte elastase inhibitor (SERPINB1), Alpha-centractin (ACTR1A), Septin-2 (SEPTIN2), Zinc finger protein 42 homolog (ZFP42), Isocitrate dehydrogenase [NADP] cytoplasmic isoform X2 (IDH1), Mitogen-activated protein kinase 1 (MAPK1), Alcohol dehydrogenase [NADP(+)] isoform X2 (AKR1A1), Poly(rC)-binding protein 1 (PCBP1), Annexin A2 (ANXA2), Aldo-keto reductase family 1 member A1 (AKR1A1), 6-phosphogluconate dehydrogenase, decarboxylating (6PGD), Citrate synthase, mitochondrial isoform X2 (CS), Creatine kinase U-type, mitochondrial isoform X1 (CKMT1), Inorganic pyrophosphatase (IPP1), ATP synthase subunit beta, mitochondrial (ATP5F1B), Actin, muscle-type (ACT), Annexin A4 (ANXA4), Proteasome activator complex subunit 2 (PSME2), Prohibitin (PHB), Keratin, type II cytoskeletal 1b (KRT77), Endoplasmic reticulum resident protein 29 (ERP29), Heat shock protein beta-1 (HSP27), Thiosulfate sulfurtransferase isoform X2 (TST), Receptor of activated protein C kinase 1 (GNB2L1), Phosphoglycerate mutase 1 (PGAM1), Triosephosphate isomerase 1 (TPI1), Myosin regulatory light chain 12B isoform X1 (MYL12B), Cofilin-1 (CFL1). ^4^ MVM—mass value matched. ^5^ MS—Mascot score. ^6^ SC—sequence coverage. ^7^ Theoretical Mr and pI values based on the Uniprot/NCBI databases. ^8^ Experimental Mr and pI values computed for each identified protein spot based on the standard 2-D markers.

**Table 5:** List of the differentially expressed protein spots identified by MALDI-TOF MS and MALDI FT-ICR MS analysis found in the cecal mucosa of nursery pigs fed a diet enriched with 4% of dried chicory root. Spot numbers correspond to those in Figure 1.

Spot No.	Gene Name ^3^	Ratio CR/C ^4^	*p*-Value
up-regulated protein spot			
30	KRT20 ^1^	2.23	<0.001 *
down-regulated protein spots			
18	PRPF19 ^1^	0.30	<0.001 *
33	ACTR3 ^1^	0.46	0.003 **
34	ALDH9A1 ^1^	0.50	0.047 **
39	G6PD ^1^	0.18	<0.001 **
44	CAP1 ^1^	0.29	<0.001 *
46	EEF1G ^1^	0.37	0.016 **
48	SERPINB1 ^1^	0.31	<0.001 *
56	ANXA2 ^2^	0.33	<0.001 **
57	AKR1A1 ^2^	0.12	<0.001 **
60	CKMT1 ^1^	0.43	0.004 **
63	ACT ^2^	0.41	0.001 *
64	ANXA4 ^2^	0.48	0.001 *
67	KRT77 ^2^	0.41	0.002 *
69	HSP27 ^2^	0.29	<0.001 *
74	TPI1 ^2^	0.13	<0.001 **

^1^ Proteins identified with the aid of the Microflex™ MALDI-TOF mass spectrometer (Bruker Daltonics, Germany). ^2^ Proteins identified with the aid of the 7T solariX™ MALDI FT-ICR mass spectrometer (SolariX 2xR, Bruker Daltonics, Germany). ^3^ Full name of proteins encoded by genes: Keratin, type I cytoskeletal 20 (KRT20), Pre-mRNA processing factor 19 (PRPF19), Actin-related protein 3 (ACTR3), 4-trimethylaminobutyraldehyde dehydrogenase1 (ALDH9A1), Glucose-6-phosphate 1-dehydrogenase isoform X2 (G6PD), Adenylyl cyclase-associated protein 1 isoform X3 (CAP1), Elongation factor 1-gamma (EEF1G), Leukocyte elastase inhibitor (SERPINB1), Annexin A2 (ANXA2), Aldo-keto reductase family 1 member A1 (AKR1A1), Creatine kinase U-type, mitochondrial isoform X1 (CKMT1), Actin, muscle-type (ACT), Annexin A4 (ANXA4), Keratin, type II cytoskeletal 1b (KRT77), Heat shock protein beta-1 (HSP27), Triosephosphate isomerase 1 (TPI1). ^4^ The average ratio of expression for protein spots found in the cecum of pigs fed a diet supplemented with 4% dried chicory root (CR) compared with the control (C) group. Values marked with * were analysed using Student’s *t*-test. Values marked with ** were analysed using Mann-Whitney test.

## Data Availability

Any data or material that support the findings of this study can be made available by the corresponding author upon request.

## Supplementary Materials

The following supporting information can be downloaded at: https://www.mdpi.com/article/10.3390/ani12131710/s1. Table S1: list of identified proteins found in the cecum of pigs annotated with the STRING database (https://string-db.org (accessed on 1 April 2022) according to their biological process; Table S2: list of identified proteins found in the cecum of pigs annotated with the STRING database (https://string-db.org (accessed on 1 April 2022) according to their molecular function; Table S3: list of identified proteins found in the cecum of pigs annotated with the STRING database (https://string-db.org (accessed on 1 April 2022) according to their cellular localization; Table S4: list of significant KEGG Pathways associated with proteins found in the cecum of pigs; Table S5: list of significant network clusters (STRING) associated with proteins found in the cecum of pigs.

## References

[1] Saffrey M.J. (2014). Aging of the mammalian gastrointestinal tract: A complex organ system. Age.

[2] Gao P., Liu Y., Le B., Qin B., Liu M., Zhao Y., Guo X., Cao G., Liu J., Li B. (2019). A comparison of dynamic distributions of intestinal microbiota between Large White and Chinese Shanxi Black pigs. Arch. Microbiol..

[3] Zhao W., Wang Y., Liu S., Huang J., Zhai Z., He C., Ding J., Wang J., Wang H., Fan W. (2015). The dynamic distribution of porcine microbiota across different ages and gastrointestinal tract segments. PLoS ONE.

[4] Celi P., Verlhac V., Pérez C.E., Schmeisser J., Kluenter A.M. (2019). Biomarkers of gastrointestinal functionality in animal nutrition and health. Anim. Feed Sci. Technol..

[5] Nwafor I.C., Shale K., Achilonu M.C. (2017). Chemical composition and nutritive benefits of chicory (*Cichorium intybus*) as an ideal complementary and/or alternative livestock feed supplement. Sci. World J..

[6] Mensink M.A., Frijlink H.W., van der Voort M., Hinrichs W.L. (2015). Inulin, a flexible oligosaccharide I: Review of its physicochemical characteristics. Carbohydr. Polym..

[7] Den Besten G., van Eunen K., Groen A.K., Venema K., Reijngoud D.J., Bakker B.M. (2013). The role of short-chain fatty acids in the interplay between diet, gut microbiota, and host energy metabolism. J. Lipid Res..

[8] Lepczyński A., Herosimczyk A., Ożgo M., Barszcz M., Taciak M., Skomiał J. (2019). Modification of ileal proteome in growing pigs by dietary supplementation with inulin or dried chicory root. J. Anim. Feed Sci..

[9] Lepczyński A., Herosimczyk A., Ożgo M., Marynowska M., Pawlikowska M., Barszcz M., Taciak M., Skomiał J. (2017). Dietary chicory root and chicory inulin trigger changes in energetic metabolism, stress prevention and cytoskeletal proteins in the liver of growing pigs—A proteomic study. J. Anim. Physiol. Anim. Nutr..

[10] Lepczynski A., Herosimczyk A., Ozgo M., Skomial J., Taciak M., Barszcz M., Berezecka N. (2015). Dietary supplementation with dried chicory root triggers changes in the blood serum proteins engaged in the clotting process and the innate immune response in growing pigs. J. Physiol. Pharmacol..

[11] Yasuda K., Maiorano R., Welch R.M., Miller D.D., Lei X.G. (2007). Cecum is the major degradation site of ingested inulin in young pigs. J. Nutr..

[12] Patterson J.K., Yasuda K., Welch R.M., Miller D.D., Lei X.G. (2010). Supplemental dietary inulin of variable chain lengths alters intestinal bacterial populations in young pigs. J. Nutr..

[13] Barszcz M., Taciak M., Skomiał J. (2018). Influence of different inclusion levels and chain length of inulin on microbial ecology and the state of mucosal protective barrier in the large intestine of young pigs. Anim. Prod. Sci..

[14] Paßlack N., Al-samman M., Vahjen W., Männer K., Zentek J. (2012). Chain length of inulin affects its degradation and the microbiota in the gastrointestinal tract of weaned piglets after a short-term dietary application. Livest. Sci..

[15] Loh G., Eberhard M., Brunner R.M., Hennig U., Kuhla S., Kleessen B., Metges C.C. (2006). Inulin alters the intestinal microbiota and short-chain fatty acid concentrations in growing pigs regardless of their basal diet. J. Nutr..

[16] Pouille C.L., Ouaza S., Roels E., Behra J., Tourret M., Molinié R., Fontaine J.-X., Mathiron D., Gagneul D., Taminiau B. (2022). Chicory: Understanding the effects and effectors of this functional food. Nutrients.

[17] Lepczyński A., Herosimczyk A., Barszcz M., Ożgo M., Michałek K., Grabowska M., Tuśnio A., Szczerbińska D., Skomiał J. (2021). Diet supplemented either with dried chicory root or chicory inulin significantly influence kidney and liver mineral content and antioxidative capacity in growing pigs. Animal.

[18] Fouhse J.M., Zijlstra R.T., Willing B.P. (2016). The role of gut microbiota in the health and disease of pigs. Anim. Front..

[19] Ghosh S., Whitley C.S., Haribabu B., Jala V.R. (2021). Regulation of intestinal barrier function by microbial metabolites. Cell Mol. Gastroenterol. Hepatol..

[20] Herosimczyk A., Lepczyński A., Ożgo M., Tuśnio A., Taciak M., Barszcz M. (2020). Effect of dietary inclusion of 1% or 3% of native chicory inulin on the large intestinal mucosa proteome of growing pigs. Animal.

[21] Barmpatsalou V., Dubbelboer I.R., Rodler A., Jacobson M., Karlsson E., Pedersen B.L., Bergström C.A.S. (2021). Physiological properties, composition and structural profiling of porcine gastrointestinal mucus. Eur. J. Pharm. Biopharm..

[22] AOAC International (2011). Official Methods of Analysis of AOAC International.

[23] Pink M., Verma N., Rettenmeier A.W., Schmitz-Spanke S. (2010). CBB staining protocol with higher sensitivity and mass spectrometric compatibility. Electrophoresis.

[24] Ozgo M., Lepczynski A., Robak P., Herosimczyk A., Marynowska M. (2019). The current proteomic landscape of the porcine liver. J. Physiol. Pharmacol..

[25] Szklarczyk D., Gable A.L., Lyon D., Junge A., Wyder S., Huerta-Cepas J., Simonovic M., Doncheva N.T., Morris J.H., Bork P. (2019). STRING v11: Protein-protein association networks with increased coverage, supporting functional discovery in genome-wide experimental datasets. Nucleic Acids Res..

[26] Tröscher-Mußotter J., Tilocca B., Stefanski V., Seifert J. (2019). Analysis of the bacterial and host proteins along and across the porcine gastrointestinal tract. Proteomes.

[27] Kiseleva O., Ponomarenko E., Poverennaya E. (2020). Empowering shotgun mass spectrometry with 2DE: A HepG2 study. Int. J. Mol. Sci..

[28] Suzuki T. (2020). Regulation of the intestinal barrier by nutrients: The role of tight junctions. Anim. Sci. J..

[29] Yang S., Yu M. (2021). Role of goblet cells in intestinal barrier and mucosal immunity. J. Inflamm. Res..

[30] Johansson M.E., Thomsson K.A., Hansson G.C. (2009). Proteomic analyses of the two mucus layers of the colon barrier reveal that their main component, the Muc2 mucin, is strongly bound to the Fcgbp protein. J. Proteome Res..

[31] Wang H., Xu R., Zhang H., Su Y., Zhu W. (2020). Swine gut microbiota and its interaction with host nutrient metabolism. Anim. Nutr..

[32] Van der Wielen N., Moughan P.J., Mensink M. (2017). Amino acid absorption in the large intestine of humans and porcine models. J. Nutr..

[33] Rodgers L.S., Fanning A.S. (2011). Regulation of epithelial permeability by the actin cytoskeleton. Cytoskeleton.

[34] Diao H., Jiao A.R., Yu B., Mao X.B., Chen D.W. (2019). Gastric infusion of short-chain fatty acids can improve intestinal barrier function in weaned piglets. Genes Nutr..

[35] Liu H., Wang J., He T., Becker S., Zhang G., Li D., Ma X. (2018). Butyrate: A double-edged sword for health?. Adv. Nutr..

[36] Darcy-Vrillon B., Cherbuy C., Morel M.T., Durand M., Duée P.H. (1996). Short chain fatty acid and glucose metabolism in isolated pig colonocytes: Modulation by NH4+. Mol. Cell. Biochem..

[37] Liu H., Ivarsson E., Dicksved J., Lundh T., Lindberg J.E. (2012). Inclusion of chicory (*Cichorium intybus* L.) in pigs' diets affects the intestinal microenvironment and the gut microbiota. Appl. Environ. Microbiol..

[38] Polari L., Alam C.M., Nyström J.H., Heikkilä T., Tayyab M., Baghestani S., Toivola D.M. (2020). Keratin intermediate filaments in the colon: Guardians of epithelial homeostasis. Int. J. Biochem. Cell Biol..

[39] Zhou Q., Cadrin M., Herrmann H., Chen C.H., Chalkley R.J., Burlingame A.L., Omary M.B. (2006). Keratin 20 serine 13 phosphorylation is a stress and intestinal goblet cell marker. J. Biol. Chem..

[40] Chan C.W., Wong N.A., Liu Y., Bicknell D., Turley H., Hollins L., Miller C.J., Wilding J.L., Bodmer W.F. (2009). Gastrointestinal differentiation marker cytokeratin 20 is regulated by homeobox gene CDX1. Proc. Natl. Acad. Sci. USA.

[41] Wang Q., Zhou Y., Rychahou P., Fan T.W., Lane A.N., Weiss H.L., Evers B.M. (2017). Ketogenesis contributes to intestinal cell differentiation. Cell Death Differ..

[42] Kien C.L., Schmitz-Brown M., Solley T., Sun D., Frankel W.L. (2006). Increased colonic luminal synthesis of butyric acid is associated with lowered colonic cell proliferation in piglets. J. Nutr..

[43] Herosimczyk A., Lepczyński A., Ożgo M., Barszcz M., Marynowska M., Tuśnio A., Taciak M., Markulen A., Skomiał J. (2018). Proteome changes in ileal mucosa of young pigs resulting from different levels of native chicory inulin in the diet. J. Anim. Feed Sci..

[44] Uchiyama K., Naito Y., Takagi T., Mizushima K., Hirai Y., Hayashi N., Harusato A., Inoue K., Fukumoto K., Yamada S. (2012). Serpin B1 protects colonic epithelial cell via blockage of neutrophil elastase activity and its expression is enhanced in patients with ulcerative colitis. Am. J. Physiol. Gastrointest. Liver Physiol..

[45] Duncan R., Carpenter B., Main L.C., Telfer C., Murray G.I. (2008). Characterisation and protein expression profiling of annexins in colorectal cancer. Br. J. Cancer.

[46] Huang C.Y., Wei P.L., Chen W.Y., Chang W.C., Chang Y.J. (2018). Silencing heat shock protein 27 inhibits the progression and metastasis of colorectal cancer (CRC) by maintaining the stability of stromal interaction molecule 1 (STIM1) proteins. Cells.

[47] Janda K., Gutowska I., Geszke-Moritz M., Jakubczyk K. (2021). The common chicory (*Cichorium intybus* L.) as a source of extracts with health-promoting properties—A review. Molecules.

