# Supplementary material for: Dietary Inclusion of Dried Chicory Root Affects Cecal Mucosa Proteome of Nursery Pigs
Source: Animals (Basel). 2022 Jul 1;12(13):1710. doi: 10.3390/ani12131710 (PMC9264899; doi:10.3390/ani12131710)
Supplement: Supplementary file 1 [file animals-12-01710-s001.zip › animals-1740817-supplementary.pdf]

Supplementary Table S1. List of identified proteins found in the cecum of pigs annotated with the STRING database (https://string-db.org) according to their biological process. For each biological process included, the corresponding Gene Ontology (GO) biological process ID and its description, the number of gene count and its identity as well as the level of significance are reported. Full name of identified proteins along with the corresponding genes can be found in Table 1.

Biological process ID	Biological process description	Gene count	P-value	Matching proteins in the network
GO:0009987	cellular process	18	6.55×10^{-9}	TUBA1B, TPI1, HSPCA, CKMT1, VCP, ALDH9A1, DST, HSP27, TF, MSN, EEF1G, C3, CALR, KRT20, HSP70.2, GNB2L1, MX1, CFL1
GO:0065007	biological regulation	15	1.06×10^{-7}	SERPINB1, PSME2, HSPCA, ANXA2, LMNA, DSTN, HSP27, TF, C3, CALR, KRT20, HSP70.2, GNB2L1, MX1, CFL1
GO:0006457	protein folding	4	1.13×10^{-5}	HSPCA, HSP27, CALR, HSP70.2
GO:0007010	cytoskeleton organization	5	5.27×10^{-5}	TUBA1B, DSTN, CALR, KRT20, CFL1
GO:0016043	cellular component organization	7	0.00012	TUBA1B, VCP, DSTN, CALR, KRT20, MX1, CFL1
GO:0050790	regulation of catalytic activity	6	0.00023	SERPINB1, PSME2, HSPCA, ANXA2, C3, GNB2L1
GO:0052548	regulation of endopeptidase activity	4	0.00040	SERPINB1, PSME2, C3, GNB2L1
GO:0051171	regulation of nitrogen compound metabolic process	7	0.00043	SERPINB1, PSME2, HSPCA, HSP27, C3, CALR, GNB2L1
GO:0051049	regulation of transport	5	0.00050	C3, CALR, KRT20, GNB2L1, MX1
GO:0031323	regulation of cellular metabolic process	7	0.00053	SERPINB1, PSME2, HSPCA, HSP27, C3, CALR, GNB2L1
GO:0046907	intracellular transport	4	0.00053	VCP, HSP27, CALR, MX1
GO:0051130	positive regulation of cellular component organization	4	0.00053	DSTN, CALR, HSP70.2, GNB2L1
GO:0051128	regulation of cellular component organization	5	0.00063	DSTN, CALR, HSP70.2, GNB2L1, CFL1
GO:0006950	response to stress	6	0.00072	HSPCA, VCP, C3, CALR, KRT20, MX1
GO:0043086	negative regulation of catalytic activity	4	0.00086	SERPINB1, ANXA2, C3, GNB2L1
GO:0051726	regulation of cell cycle	4	0.00086	PSME2, CALR, HSP70.2, GNB2L1
GO:1901566	organonitrogen compound biosynthetic process	5	0.00086	TPI1, VCP, ALDH9A1, EEF1G, GNB2L1

GO:0032269	negative regulation of cellular protein metabolic process	4	0.0012	SERPINB1, C3, CALR, GNB2L1
GO:0097435	supramolecular fiber organization	3	0.0015	DSTN, KRT20, CFL1
GO:0065008	regulation of biological quality	6	0.0016	HSPCA, DSTN, TF, C3, CALR, GNB2L1
GO:0006414	translational elongation	2	0.0018	EEF1G, GNB2L1
GO:0030036	actin cytoskeleton organization	3	0.0018	DSTN, CALR, CFL1
GO:0030042	actin filament depolymerization	2	0.0018	DSTN, CFL1
GO:0048519	negative regulation of biological process	6	0.0018	SERPINB1, LMNA, C3, CALR, GNB2L1, MX1
GO:0044271	cellular nitrogen compound biosynthetic process	5	0.0022	TPI1, VCP, ALDH9A1, EEF1G, GNB2L1
GO:0009893	positive regulation of metabolic process	5	0.0023	PSME2 ,HSPCA, C3, CALR, GNB2L1
GO:0016197	endosomal transport	2	0.0036	VCP, MX1
GO:0008152	metabolic process	8	0.0038	TPI1, CKMT1, VCP, ALDH9A1, EEF1G, C3, AKR1A1, GNB2L1
GO:0010564	regulation of cell cycle process	3	0.0038	PSME2, CALR, HSP70.2
GO:0022603	regulation of anatomical structure morphogenesis	3	0.0046	CALR, GNB2L1, CFL1
GO:0034613	cellular protein localization	3	0.0050	LMNA, HSP27, CALR
GO:0050896	response to stimulus	7	0.0050	HSPCA, VCP, C3, CALR, KRT20, GNB2L1, MX1
GO:0006810	transport	5	0.0051	VCP, HSP27, TF, CALR, MX1
GO:1901564	organonitrogen compound metabolic process	6	0.0052	TPI1, VCP, ALDH9A1 ,EEF1G, C3, GNB2L1
GO:0033043	regulation of organelle organization	3	0.0057	DSTN, CALR, HSP70.2
GO:0044237	cellular metabolic process	7	0.0058	TPI1, CKMT1, VCP, ALDH9A1, EEF1G, C3, GNB2L1
GO:0043900	regulation of multi-organism process	3	0.0059	ANXA2, CALR, MX1
GO:0050764	regulation of phagocytosis	2	0.0059	CALR, GNB2L1
GO:0050793	regulation of developmental process	4	0.0059	LMNA, CALR, GNB2L1, CFL1
GO:0051050	positive regulation of transport	3	0.0059	C3, CALR, GNB2L1
GO:0051495	positive regulation of cytoskeleton organization	2	0.0059	DSTN, HSP70.2

GO:0016567	protein ubiquitination	2	0.0062	VCP, GNB2L1
GO:1902905	positive regulation of supramolecular fiber organization	2	0.0062	DSTN, HSP70.2
GO:0051783	regulation of nuclear division	2	0.0069	CALR, HSP70.2
GO:0061136	regulation of proteasomal protein catabolic process	2	0.0069	PSME2, GNB2L1
GO:0002376	immune system process	4	0.0075	MSN, C3, CALR, MX1
GO:0033554	cellular response to stress	3	0.0078	VCP, CALR, KRT20
GO:0034504	protein localization to nucleus	2	0.0082	LMNA, CALR
GO:0010950	positive regulation of endopeptidase activity	2	0.0090	PSME2, GNB2L1
GO:0051173	positive regulation of nitrogen compound metabolic process	4	0.0091	PSME2, HSPCA, C3, GNB2L1
GO:0030100	regulation of endocytosis	2	0.0093	CALR, GNB2L1
GO:0051094	positive regulation of developmental process	3	0.0093	LMNA, CALR, GNB2L1
GO:0010604	positive regulation of macromolecule metabolic process	4	0.0094	PSME2, C3, CALR, GNB2L1
GO:0031334	positive regulation of protein complex assembly	2	0.0100	HSP70.2, GNB2L1
GO:0048585	negative regulation of response to stimulus	3	0.0100	LMNA, CALR, GNB2L1
GO:0006417	regulation of translation	2	0.0107	CALR, GNB2L1
GO:0031325	positive regulation of cellular metabolic process	4	0.0107	PSME2, HSPCA, C3, GNB2L1
GO:0006575	cellular modified amino acid metabolic process	2	0.0115	ALDH9A1, EEF1G
GO:0031326	regulation of cellular biosynthetic process	4	0.0116	HSPCA, C3, CALR, GNB2L1
GO:0001932	regulation of protein phosphorylation	3	0.0130	HSP27, C3, GNB2L1
GO:0043901	negative regulation of multi-organism process	2	0.0130	CALR, MX1
GO:0022604	regulation of cell morphogenesis	2	0.0136	CALR, CFL1
GO:0009967	positive regulation of signal transduction	3	0.0150	C3, CALR, GNB2L1

GO:0032270	positive regulation of cellular protein metabolic process	3	0.0172	PSME2, C3, GNB2L1
GO:0007346	regulation of mitotic cell cycle	2	0.0205	PSME2, HSP70.2
GO:0010951	negative regulation of endopeptidase activity	2	0.0205	SERPINB1, C3
GO:0051716	cellular response to stimulus	5	0.0206	VCP, CALR, KRT20, GNB2L1, MX1
GO:0051223	regulation of protein transport	2	0.0220	KRT20, GNB2L1
GO:0030335	positive regulation of cell migration	2	0.0228	CALR, GNB2L1
GO:0034622	cellular protein-containing complex assembly	2	0.0237	CALR, MX1
GO:0051051	negative regulation of transport	2	0.0237	GNB2L1, MX1
GO:0019538	protein metabolic process	4	0.0274	VCP, EEF1G, C3, GNB2L1
GO:0042221	response to chemical	4	0.0282	HSPCA, VCP, GNB2L1, MX1
GO:0048583	regulation of response to stimulus	4	0.0290	LMNA, C3, CALR, GNB2L1
GO:0051186	cofactor metabolic process	2	0.0319	TPI1, EEF1G
GO:0002252	immune effector process	2	0.0327	C3, MX1
GO:0032787	monocarboxylic acid metabolic process	2	0.0377	TPI1, C3
GO:0009968	negative regulation of signal transduction	2	0.0430	CALR, GNB2L1
GO:0034645	cellular macromolecule biosynthetic process	3	0.0437	VCP,EEF1G, GNB2L1
GO:1901137	carbohydrate derivative biosynthetic process	2	0.0440	TPI1, VCP
GO:0050801	ion homeostasis	2	0.0447	TF, GNB2L1
GO:0055082	cellular chemical homeostasis	2	0.0447	TF, GNB2L1
GO:0009605	response to external stimulus	3	0.0471	C3, KRT20, MX1

Supplementary Table S2. List of identified proteins found in the cecum of pigs annotated with the STRING database (https://string-db.org) according to their molecular function. For each molecular function included, the corresponding Gene Ontology (GO) molecular function ID and its description, the number of gene count and its identity as well as the level of significance are reported. Full name of identified proteins along with the corresponding genes can be found in Table 1.

Molecular function ID	Molecular function description	Gene count	P-value	Matching proteins in the network
GO:0005488	binding	17	2.79×10^{-9}	TUBA1B, HSPCA, ANXA2, CKMT1, VCP, DSTN, HSP27, ANXA4, TF, MSN, EEF1G, C3, CALR, HSP70.2, GNB2L1, MX1, CFL1
GO:0005515	protein binding	10	4.52×10^{-6}	HSPCA, ANXA2, DSTN, HSP27, MSN, C3, CALR, GNB2L1, MX1, CFL1
GO:0030234	enzyme regulator activity	6	6.04×10^{-6}	SERPINB1, PSME2, HSPCA, ANXA2, C3, GNB2L1
GO:0043167	ion binding	10	6.04×10^{-6}	TUBA1B, HSPCA, ANXA2, CKMT1, VCP, ANXA4, TF, CALR, HSP70.2, MX1
GO:0008092	cytoskeletal protein binding	5	1.20×10^{-5}	ANXA2, DSTN, MSN, MX1, CFL1
GO:0032555	purine ribonucleotide binding	6	2.80×10^{-5}	TUBA1B, HSPCA, CKMT1, VCP, HSP70.2, MX1
GO:0003824	catalytic activity	9	3.14×10^{-5}	TUBA1B, TPI1, HSPCA, CKMT1, VCP, ALDH9A1, EEF1G, AKR1A1, MX1
GO:1901363	heterocyclic compound binding	8	3.30×10^{-5}	TUBA1B, HSPCA, CKMT1, VCP, EEF1G, CALR, HSP70.2, MX1
GO:0061134	peptidase regulator activity	4	4.14×10^{-5}	SERPINB1, PSME2, C3, GNB2L1
GO:0097159	organic cyclic compound binding	8	4.14×10^{-5}	TUBA1B, HSPCA, CKMT1, VCP, EEF1G, CALR, HSP70.2, MX1
GO:0004857	enzyme inhibitor activity	4	0.00013	SERPINB1, ANXA2, C3, GNB2L1
GO:0017111	nucleoside-triphosphatase activity	4	0.00015	TUBA1B, HSPCA, VCP, MX1
GO:0005544	calcium-dependent phospholipid binding	2	0.00039	ANXA2, ANXA4
GO:0061135	endopeptidase regulator activity	3	0.00061	SERPINB1, PSME2, C3
GO:0003779	actin binding	3	0.00075	DSTN, MSN, CFL1
GO:0005524	ATP binding	4	0.00075	HSPCA, CKMT1, VCP, HSP70.2
GO:0051082	unfolded protein binding	2	0.00087	HSPCA, CALR
GO:0008144	drug binding	4	0.0016	HSPCA, CKMT1, VCP, HSP70.2
GO:0008289	lipid binding	3	0.0016	ANXA2, VCP, ANXA4
GO:0044877	protein-containing complex binding	3	0.0022	CALR, GNB2L1, CFL1

GO:0005509	calcium ion binding	3	0.0023	ANXA2, ANXA4, CALR
GO:0005198	structural molecule activity	3	0.0045	TUBA1B, LMNA, KRT20
GO:0019904	protein domain specific binding	2	0.0056	HSPCA, GNB2L1
GO:0003924	GTPase activity	2	0.0068	TUBA1B, MX1
GO:0016887	ATPase activity	2	0.0094	HSPCA, VCP
GO:0004866	endopeptidase inhibitor activity	2	0.0104	SERPINB1, C3
GO:0005525	GTP binding	2	0.0115	TUBA1B, MX1
GO:0046872	metal ion binding	4	0.0179	ANXA2, ANXA4, TF, CALR
GO:0042803	protein homodimerization activity	2	0.0180	HSP27, GNB2L1
GO:0005102	signaling receptor binding	3	0.0278	C3, CALR, GNB2L1
GO:0003723	RNA binding	2	0.0310	EEF1G, CALR
GO:0019899	enzyme binding	2	0.0457	CALR, GNB2L1

Supplementary Table S3. List of identified proteins found in the cecum of pigs annotated with the STRING database (https://string-db.org) according to their cellular localization. For each cellular component included, the corresponding Gene Ontology (GO) cellular component ID and its description, the number of gene count and its identity as well as the level of significance are reported. Full name of identified proteins along with the corresponding genes can be found in Table 1.

Cellular component ID	Cellular component description	Gene count	P-value	Matching proteins in the network
GO:0005622	intracellular	20	1.72×10^{-12}	TUBA1B, TPI1, SERPINB1, PSME2, HSPCA, ANXA2, CKMT1, VCP, ALDH9A1, LMNA, DSTN, HSP27, MSN, EEF1G, CALR, KRT20, HSP70.2, GNB2L1, MX1, CFL1
GO:0005737	cytoplasm	18	8.45×10^{-12}	TUBA1B, TPI1, SERPINB1, PSME2, HSPCA, ANXA2, CKMT1, VCP, ALDH9A1, DSTN, HSP27, MSN, EEF1G, CALR, HSP70.2, GNB2L1, MX1, CFL1
GO:0043229	intracellular organelle	16	2.59×10^{-10}	TUBA1B, HSPCA, ANXA2, CKMT1, VCP, LMNA, DSTN, HSP27, MSN, EEF1G, CALR, KRT20, HSP70.2, GNB2L1, MX1, CFL1
GO:0005856	cytoskeleton	8	3.71×10^{-9}	TUBA1B, LMNA, DSTN, HSP27, MSN, KRT20, HSP70.2, CFL1
GO:0043232	intracellular non-membrane-bounded organelle	10	3.71×10^{-9}	TUBA1B, VCP, LMNA, DSTN, HSP27, MSN, KRT20, HSP70.2, GNB2L1, CFL1
GO:0005634	nucleus	8	5.35×10^{-6}	HSPCA, VCP, LMNA, HSP27, EEF1G, CALR, GNB2L1, CFL1
GO:0043227	membrane-bounded organelle	11	5.35×10^{-6}	HSPCA, ANXA2, CKMT1, VCP, LMNA, HSP27, EEF1G, CALR, GNB2L1, MX1, CFL1
GO:0099513	polymeric cytoskeletal fiber	3	0.00075	TUBA1B, LMNA, KRT20
GO:0005882	intermediate filament	2	0.0010	LMNA, KRT20
GO:0015630	microtubule cytoskeleton	3	0.0010	TUBA1B, HSP27, HSP70.2
GO:0031967	organelle envelope	4	0.0010	CKMT1, LMNA, CALR, MX1
GO:0071944	cell periphery	6	0.0010	HSPCA, DSTN, MSN, CALR, GNB2L1, CFL1
GO:0016020	membrane	7	0.0021	HSPCA, CKMT1, MSN, CALR, GNB2L1, MX1, CFL1
GO:0070013	intracellular organelle lumen	4	0.0021	LMNA, CALR, GNB2L1, CFL1
GO:0031253	cell projection membrane	2	0.0031	MSN, CFL1

GO:0005829	cytosol	4	0.0039	TPI1, VCP, CALR, GNB2L1
GO:0005886	plasma membrane	5	0.0040	HSPCA, MSN, CALR, GNB2L1, CFL1
GO:0042470	melanosome	2	0.0056	HSPCA, ANXA2
GO:0005635	nuclear envelope	2	0.0057	LMNA, CALR
GO:0031981	nuclear lumen	3	0.0063	LMNA, GNB2L1, CFL1
GO:0005576	extracellular region	5	0.0067	SERPINB1, ANXA2, TF, C3, CALR
GO:0015629	actin cytoskeleton	2	0.0105	DSTN, CFL1
GO:0012505	endomembrane system	4	0.0126	VCP, LMNA, CALR, MX1
GO:0005783	endoplasmic reticulum	3	0.0130	VCP, CALR, MX1
GO:0005739	mitochondrion	3	0.0183	CKMT1, GNB2L1, MX1
GO:0005654	nucleoplasm	2	0.0302	LMNA, GNB2L1
GO:1990904	ribonucleoprotein complex	2	0.0412	CALR, GNB2L1
GO:0031966	mitochondrial membrane	2	0.0424	CKMT1, MX1

Supplementary Table S4. List of significant KEGG Pathways associated with proteins found in the cecum of pigs. For each pathway included, the corresponding ID and its description, the number of gene count and its identity as well as the level of significance are reported. Full name of identified proteins along with the corresponding genes can be found in Table 1.

KEGG Pathway ID	KEGG Pathway description	Gene count	P-value	Matching proteins in the network
ssc00010	Glycolysis / Gluconeogenesis	4	0.0013	TPI1, ALDH9A1, PGAM1, AKR1A1
ssc04141	Protein processing in endoplasmic reticulum	5	0.0016	HSPCA, VCP, ERP29, CALR, HSP70.2
ssc01200	Carbon metabolism	4	0.0042	TPI1, PGAM1, G6PD, IDH1
ssc01100	Metabolic pathways	10	0.0060	TST, TPI1, CKMT1, ALDH9A1, GFPT1, PGAM1, G6PD, AKR1A1, IDH1, UGDH
ssc04530	Tight junction	4	0.0066	TUBA1B, MYL12B, MSN, ACTR3
ssc05230	Central carbon metabolism in cancer	3	0.0066	MAPK1, PGAM1, G6PD
ssc01230	Biosynthesis of amino acids	3	0.0071	TPI1, PGAM1, IDH1
ssc00053	Ascorbate and aldarate metabolism	2	0.0090	ALDH9A1, UGDH
ssc00040	Pentose and glucuronate interconversions	2	0.0111	AKR1A1, UGDH
ssc04810	Regulation of actin cytoskeleton	4	0.0111	MYL12B, MAPK1, MSN, CFL1
ssc04145	Phagosome	3	0.0333	TUBA1B, C3, CALR
ssc04210	Apoptosis	3	0.0333	TUBA1B, LMNA, MAPK1
ssc00330	Arginine and proline metabolism	2	0.0407	CKMT1, ALDH9A1
ssc00480	Glutathione metabolism	2	0.0407	G6PD, IDH1
ssc00520	Amino sugar and nucleotide sugar metabolism	2	0.0424	GFPT1, UGDH

Supplementary Table S5. List of significant local network clusters (STRING) associated with proteins found in the cecum of pigs. For each cluster included, the corresponding ID and its description, the number of gene count and its identity as well as the level of significance are reported. Full name of identified proteins along with the corresponding genes can be found in Table 1.

Network cluster ID	Network cluster description	Gene count	P-value	Matching proteins in the network
CL:23712	mixed, incl. Carbon metabolism, and Amino sugar and nucleotide sugar metabolism	8	2.54×10^{-6}	CS, TPI1, GFPT1, PGAM1, G6PD, IDH1, UGDH, ENO1
CL:23718	Carbon metabolism, and lactate/malate dehydrogenase, NAD binding domain	6	6.96×10^{-6}	CS, TPI1, PGAM1, G6PD, IDH1, ENO1
CL:23803	Pentose phosphate pathway, and Glycolysis	4	0.00010	TPI1, PGAM1, G6PD, ENO1
CL:23809	Glycolysis, and Enolase, N-terminal domain	3	0.00022	TPI1, PGAM1, ENO1
CL:19479	TCP-1/cpn60 chaperonin family, and translation elongation factor activity	3	0.00042	CCT7, EEF1G, CCT5
CL:24310	mixed, incl. glyceraldehyde-3-phosphate dehydrogenase (NAD+) (non-phosphorylating) activity, and Acyl-CoA reductase (LuxC)	2	0.0037	ALDH9A1, AKR1A1
CL:19481	Chaperonin TCP-1, conserved site	2	0.0081	CCT7, CCT5
CL:18094	DnaJ C terminal domain, and Stress response	2	0.0111	HSPCA, HSP70.2
CL:18082	mixed, incl. Protein processing in endoplasmic reticulum, and DnaJ domain	4	0.0122	HSPCA, ERP29, CALR, HSP70.2
CL:33012	Intermediate filament protein	2	0.0142	KRT20, KRT77
CL:4830	mixed, incl. RHO GTPases Activate WASPs and WAVEs, and Cofilin/tropomyosin-type actin-binding protein	3	0.0142	DSTN, ACTR3, CFL1
CL:23721	Tricarboxylic acid cycle, and Citrate synthase, C-terminal domain	2	0.0212	CS, IDH1
CL:18326	mixed, incl. Calnexin/calreticulin cycle, and ERO1-like superfamily	2	0.0259	ERP29, CALR
CL:21078	mRNA Splicing - Major Pathway	3	0.0323	PRPF19, PCBP1, HNRNPK
CL:23942	Amino sugar and nucleotide sugar metabolism, and Glucose-6-phosphatase	2	0.0358	GFPT1, UGDH
CL:4833	RHO GTPases Activate WASPs and WAVEs, and Profilin	2	0.0358	ACTR3, CFL1
CL:24104	mixed, incl. Metabolism of amino acids and derivatives, and beta-Alanine metabolism	3	0.0413	TST, ALDH9A1, AKR1A1
